# Clinicopathological Implications of ASAP1 Expression in Hepatocellular Carcinoma

**DOI:** 10.3389/pore.2022.1610635

**Published:** 2022-08-30

**Authors:** Seongsik Bang, Seungyun Jee, Hwangkyu Son, Hyebin Cha, Jongmin Sim, Yeseul Kim, Hosub Park, Jaekyung Myung, Hyunsung Kim, Seungsam Paik

**Affiliations:** ^1^ Department of Pathology, Seoul Hospital, Hanyang University College of Medicine, Seoul, South Korea; ^2^ Department of Pathology, Anam Hospital, Korea University College of Medicine, Seoul, South Korea

**Keywords:** immunohistochemistry, prognosis, hepatocellular carcinoma, ArfGAP with SH3 domain ankyrin repeat and PH domain 1, ASAP1

## Abstract

**Background:** The expression of ArfGAP with SH3 domain ankyrin repeat and PH domain 1 (ASAP1) is increased in various types of cancer, showing potential as a prognostic marker. The clinicopathological implications of ASAP1 expression in patients with hepatocellular carcinoma (HCC) remain unclear. We thus investigated the clinicopathological significance and prognostic effect of ASAP1 expression in HCC patients.

**Materials and Methods:** ASAP1 expression was assessed in 149 HCC tissue samples using immunohistochemistry (IHC). The associations between ASAP1 expression and clinicopathological characteristics were analyzed. The prognostic effect of ASAP1 expression in patients with HCC was evaluated based on survival analyses and confirmed using a web-based tool.

**Results:** ASAP1 expression was observed in the cytoplasm of tumor cells. High ASAP1 expression was observed in 89 (59.7%) of 149 cases. High ASAP1 expression was significantly associated with male patients (*p* = 0.018), higher histological grade (*p* = 0.013), vessel invasion (*p* = 0.021), and higher stage (*p* = 0.020). High ASAP1 expression was associated with shorter overall survival (OS; *p* = 0.041) and recurrence-free survival (RFS; *p* = 0.008) based on Kaplan-Meier survival analyses. Web-based analysis using Kaplan-Meier (KM) plotter showed high mRNA ASAP1 expression to be associated with short OS (*p* = 0.001).

**Conclusion:** High ASAP1 expression was associated with aggressive clinicopathological characteristics and poor clinical outcomes in patients with HCC. ASAP1 can be considered a prognostic biomarker in HCC patients.

## Introduction

Primary liver cancer is a common type of cancer and the leading cause of cancer-related deaths worldwide. According to the GLOBOCAN database, primary liver cancer was the sixth most frequently diagnosed cancer and the fourth most common cause of cancer-related deaths in 2020 (1). Hepatocellular carcinoma (HCC) accounts for the majority (75%–90%) of liver cancers, and the incidence varies by region depending on etiological factors such as hepatitis B virus (HBV), hepatitis C virus (HCV), and alcohol intake (2,3). For treatment of HCC, classical treatment modalities (locoregional therapies and surgical resection) have been mainly considered; recently, multikinase inhibitors and immune agents were included as treatment options based on the molecular mechanisms of cancer (4). However, patients with advanced HCC still have a poor prognosis, and research to discover clinically useful predictive and prognostic biomarkers is essential.

ArfGAP with SH3 domain ankyrin repeat and PH domain 1 (ASAP1/AMAP1/DDEF1) is a downstream molecule of the ADP-ribosylation factor (ARF) family and is involved in various biological functions such as membrane trafficking and cell movement (5-7). The role of ASAP1 in cancer has been investigated in numerous studies. Overexpression of ASAP1 was identified in cancer cell lines including uveal melanoma, prostate cancer, breast cancer, and colorectal cancer, indicating that ASAP1 promotes cancer invasion and metastasis (8-12). Similarly, in studies using cancer tissue obtained from patients, ASAP1 acted as an oncogene, and its potential as a prognostic marker was recognized. Increased ASAP1 expression in different types of cancer, including colon cancer, head and neck squamous cell carcinoma, epithelial ovarian cancer, renal cancer, pancreas cancer, gastric cancer, and triple-negative breast cancer, was associated with poor prognosis and aggressive clinical features (12-20).

Wang et al. found the ASAP1 mRNA level to be increased in human HCC tissues compared with non-tumor tissues. They demonstrated that ASAP1 was co-expressed with eukaryotic translation initiation factor 5B (eIF5B) and suggested that eIF5B might promote proliferation and metastasis through increased ASAP1 expression (21). However, the effects on patient prognosis and clinicopathological parameters of ASAP1 expression have not been clearly identified. Therefore, in the present study, we investigated the correlations between ASAP1 expression and clinicopathological characteristics in human HCC tissues and their prognostic significance through immunohistochemical (IHC) staining.

## Materials and Methods

### Patient Selection and Data Collection

The present study included liver cancer patients who underwent surgical treatment at Hanyang University Hospital (Seoul, Korea) between January 1991 and September 2013, and 166 patients with complete follow-up data were retrospectively enrolled. We excluded 17 cases with unavailable tumor tissue or who underwent preoperative treatment (embolization or ablation). Finally, 149 cases were included in the present study. To compare the ASAP1 expression between HCC and normal liver parenchyma, we additionally obtained 12 normal liver tissues. Two pathologists (HK and SP) reviewed all tissue slides and pathology reports used at diagnosis. We obtained tumor size, histological grade, vessel invasion, perineural invasion, tumor focality, proliferation index, and pathological stage. Edmondson and Steiner grading system was used to evaluate histological grade, and the pathological stage was based on the 8th edition of the American Joint Committee on Cancer (AJCC). The proliferation index was evaluated using IHC staining for Ki-67 protein. Medical records were reviewed to obtain clinical characteristics, including patient age, sex, etiological factors, underlying diseases, alpha-fetoprotein (AFP) level, Child-Pugh class, Barcelona Clinic Liver Cancer (BCLC) stage, and follow-up data. The study was conducted in accordance with the Declaration of Helsinki, and approved by the Institutional Review Board of the Hanyang University Hospital (IRB file No. HYUH 2015-12-017). The requirement for informed consent was waived.

### Tissue Microarray Construction

A manual tissue microarrayer (Unitma, Seoul, Korea) and formalin-fixed paraffin-embedded tissues were used for tissue microarray (TMA) construction. We selected a representative portion of the tumor that contained almost no necrosis by light microscopy and obtained tissue cores (2.0 mm in diameter, 1 core per case) from the corresponding donor block. Then, the tissue cores were transferred to a recipient block consisting of 8 × 5 samples.

### Immunohistochemistry

IHC staining for ASAP1 was performed with 4-μm-thick sections using the Benchmark XT automated staining system (Ventana Medical Systems, Tucson, AZ, United States). Heat-induced epitope retrieval is performed with CC1 Tris-EDTA buffer (Ventana Medical Systems, Tucson, AZ, United States) at 100°C for 80 min. Subsequent procedures were performed with OptiView DAB IHC Detection Kit (Ventana Medical Systems, Tucson, AZ, United States). For blocking endogenous peroxidase, OptiView Peroxidase Inhibitor was used, and Optiview DAB was used as Chromogen. Counterstaining was performed using modified Mayer’s hematoxylin (Hematoxylin II). The recombinant anti-ASAP1 antibody (ab125729, Abcam, Cambridge, UK; diluted 1:200) was used as the primary antibody. IHC staining for Ki-67 (M7240, Dako, Glostrup, Denmark) was performed using the Bond-Max automated immunostainer (Leica Biosystems, Wetzlar, Germany).

### Assessment of Immunohistochemistry

As reported in previous studies (10, 12, 14-16, 19), we considered cytoplasmic staining of the tumor cells to be positive. All IHC sections were evaluated using light microscopy by two pathologists (SB and SP) without access to clinical data. For semi-quantitative assessment, the immunoreactive score (IRS) was calculated as the product of staining intensity and proportion of positive cells. The intensity of staining was categorized as 0–3 (0: negative, 1: weak, 2: moderate, and 3: strong), and the proportion of staining was assigned as 0 (0%), 1 (1%–25%), 2 (26%–50%), 3 (51–75%), and 4 (>75%). A receiver operating characteristics (ROC) curve analysis was used to determine the optimal cutoff value, and we divided the cases into low and high expression groups (IRS ≤4 and IRS >4, respectively).

### Web-Based Survival Analysis

Kaplan-Meier plotter (KM plotter, http://kmplot.com/analysis/) was used to predict the association between the mRNA expression level of ASAP1 and the survival of HCC patients. KM plotter is a web-based tool based on The Cancer Genome Atlas (TCGA) data and includes 364 HCC cases with available follow-up data (22). Using this tool, HCC cases were divided into two groups (high or low expression), and the effect of ASAP1 expression on the prognosis was estimated through survival analysis (23).

### Statistical Analyses

We used SPSS software version 25.0 (IBM, Armonk, NY, United States) for statistical analysis. ASAP1 expression in HCC and normal liver tissue was compared using the Mann-Whitney U test. The correlations between ASAP1 expression and clinicopathological characteristics were analyzed using Pearson’s chi-square (χ2) or Fisher’s exact test. Overall survival (OS) and recurrence-free survival (RFS) of HCC patients were evaluated using the Kaplan-Meier method with log-rank test. The Cox proportional hazards model was used to determine the significant prognostic factors. We used univariate analysis to determine the effect of individual parameters (ASAP1 expression and other clinicopathological factors) on OS or RFS. Then, multivariate analysis was used to analyze how prognostic factors jointly affect. A two-tailed *p*-value < 0.05 was considered statistically significant.

## Results

### Baseline Characteristics

The median age of the patients was 55 years (range: 28–87 years), and the male/female ratio was 3.02:1. Among etiological factors, HBV (123 cases, 82.6%) was the most common, followed by alcohol intake (10 cases, 6.7%) and HCV (7 cases, 4.7%). Based on the Edmondson and Steiner grading system, 12 cases (8.1%) were grade 1, 54 cases (36.2%) were grade 2, 70 cases (47.0%) were grade 3, and 13 cases (8.7%) were grade 4. Vessel invasion was identified in 59 cases (39.6%). Most patients were stage I or II (126 cases, 84.6%) according to the 8th AJCC staging system. Among patients, 65 (43.6%) received only curative surgery, 5 (3.4%) received radiofrequency ablation, 68 (45.6%) received chemoembolization, 6 (4.0%) received systemic chemotherapy, and 5 (3.4%) received combined treatment. The clinicopathological characteristics of patients with HCC are summarized in Table 1.

**TABLE 1:** Clinicopathological characteristics of patients with HCC (*n* = 149).

Clinicopathological characteristics	Case no. (%)
Age, median (range, years)	55 (28–87)
Sex	
Male	112 (75.2%)
Female	37 (24.8%)
Etiological factors	
HBV	123 (82.6%)
HCV	7 (4.7%)
Alcohol intake	10 (6.7%)
None	9 (6.0%)
Underlying diseases	
Cirrhosis	131 (87.9%)
Hepatitis	15 (10.1%)
None	3 (2.0%)
AFP level	
<400	97 (65.1%)
≥400	34 (22.8%)
Not reported	18 (12.1%)
Child-Pugh class	
A	138 (92.6%)
B	11 (7.4%)
BLCL stage	
A	85 (57.0%)
B	5 (3.4%)
C	59 (39.6%)
Tumor size, mean (range, cm)	4.5 (0.7–22.0)
Histological grade (Edmondson and Steiner grading system)	
Grade 1	12 (8.1%)
Grade 2	54 (36.2%)
Grade 3	70 (47.0%)
Grade 4	13 (8.7%)
Vessel invasion	
Present	59 (39.6%)
Not identified	90 (60.4%)
Perineural invasion	
Present	4 (2.7%)
Not identified	145 (97.3%)
Tumor focality	
Single	125 (83.9%)
Multiple	24 (16.1%)
Proliferation index (Ki-67 index)	
<1%	114 (76.5%)
≥1%	35 (23.5%)
Stage (AJCC 8th edition)	
IA	22 (14.8%)
IB	59 (39.6%)
II	45 (30.2%)
IIIA	6 (4.0%)
IIIB	17 (11.4%)
Additional treatments	
None	65 (43.6%)
Radiofrequency ablation	5 (3.4%)
Chemoembolization	68 (45.6%)
Systemic chemotherapy	6 (4.0%)
Combined	5 (3.4%)

HCC, hepatocellular carcinoma; HBV, hepatitis B virus; HCV, hepatitis C virus; AFP, alpha-fetoprotein; BLCL, Barcelona Clinic Liver Cancer; AJCC, American Joint Committee on Cancer.

### ASAP1 Expression in HCC and Normal Liver Tissue

We evaluated ASAP1 expression in 12 normal liver tissues and 149 HCC tissues. The mean IRS of ASAP1 expression was 2.50 (±1.88) in normal liver tissues and 6.26 (±3.16) in HCC tissues. The mean rank of IRS in HCC was significantly higher than that of normal liver tissues (*p* = 0.001, Mann-Whitney U test).

### Correlations Between ASAP1 Expression and Clinicopathological Characteristics

**FIGURE 1:**
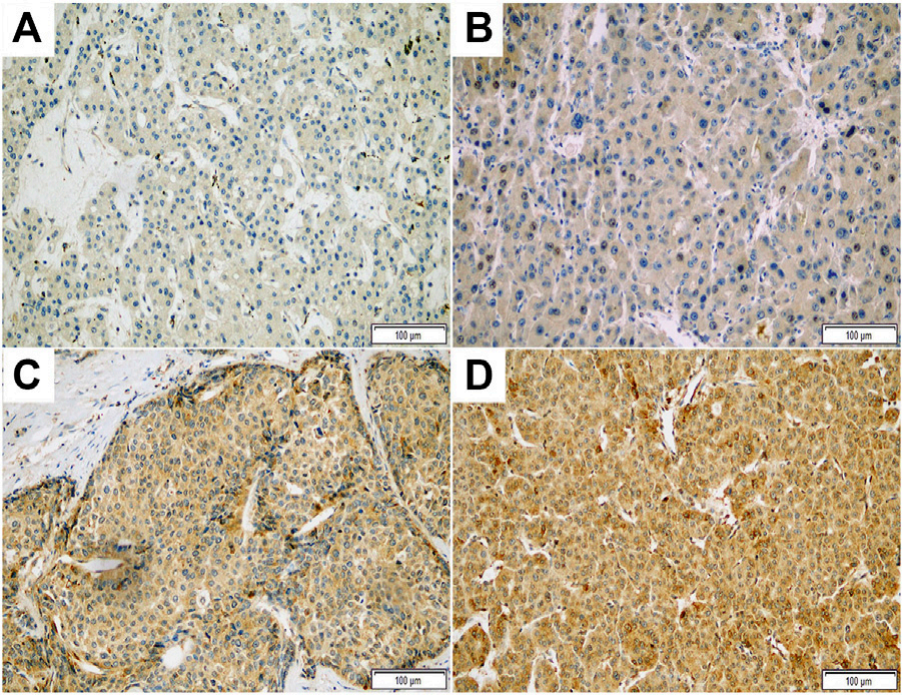
Representative photomicrographs of immunohistochemical staining with ASAP1 in hepatocellular carcinoma (x200). Negative **(A)**, weak **(B)**, moderate **(C)**, and strong **(D)** cytoplasmic expression.

Representative photomicrographs of ASAP1 IHC are presented in Figure 1, and we presented raw data including IHC results and clinicopathological characteristics for each case as Supplementary Material. Among the 149 cases, 89 (59.7%) were classified as high ASAP1 expression. High ASAP1 expression was significantly associated with male patients (*p* = 0.018), higher histological grade (*p* = 0.013), vessel invasion (*p* = 0.021), and higher stage (*p* = 0.020). High ASAP1 expression was identified more frequently in cirrhotic HCC than in non-cirrhotic HCC, however, it was not statistically significant (*p* = 0.054). The correlations between ASAP1 expression and clinicopathological characteristics are summarized in Table 2.

**TABLE 2:** Correlations between ASAP1 expression and clinicopathological characteristics in patients with HCC (*n* = 149).

Variables	ASAP1 expression		*p*-value
	Low expression (%) (*n* = 60)	High expression (%) (n = 89)	
Age			0.550
<60 years	39 (38.6%)	62 (61.4%)	
≥60 years	21 (51.4%)	27 (48.6%)	
Sex			0.018
Female	21 (56.8%)	16 (43.2%)	
Male	39 (34.8%)	73 (65.2%)	
Underlying diseases			0.054
Non‐cirrhotic HCC	11 (61.1%)	7 (38.9%)	
Cirrhotic HCC	49 (37.4%)	82 (62.6%)	
AFP level^a^			0.307
<400	44 (45.4%)	53 (54.6%)	
≥400	12 (35.3%)	22 (64.7%)	
Tumor size			0.793
≤5 cm	43 (41.0%)	62 (59.0%)	
>5 cm	17 (38.6%)	27 (61.4%)	
Histological grade			0.013
Grade 1 or 2	34 (51.5%)	32 (48.5%)	
Grade 3 or 4	26 (31.3%)	57 (68.7%)	
Vessel invasion			0.021
Not identified	43 (47.8%)	47 (52.2%)	
Present	17 (28.8%)	42 (71.2%)	
Perineural invasion			0.649^b^
Not identified	59 (40.7%)	86 (59.3%)	
Present	1 (25.0%)	3 (75.0%)	
Tumor focality			0.226
Single	53 (42.4%)	72 (57.6%)	
Multiple	7 (29.2%)	17 (70.8%)	
Proliferation index (Ki-67 index)			0.844
<1%	45 (39.5%)	69 (60.5%)	
≥1%	15 (42.9%)	20 (57.1%)	
Stage (AJCC 8th edition)			0.020^b^
I or II	56 (44.4%)	70 (55.6%)	
III	4 (17.4%)	19 (82.6%)	

a AFP level, 18 cases missed.

b Fisher’s exact test.

Acronyms: HCC, hepatocellular carcinoma; AFP, alpha-fetoprotein; ASAP1, ArfGAP with SH3 domain ankyrin repeat and PH domain 1; AJCC, American Joint Committee on Cancer.

### Prognostic Implication of ASAP1 Expression

**FIGURE 2:**
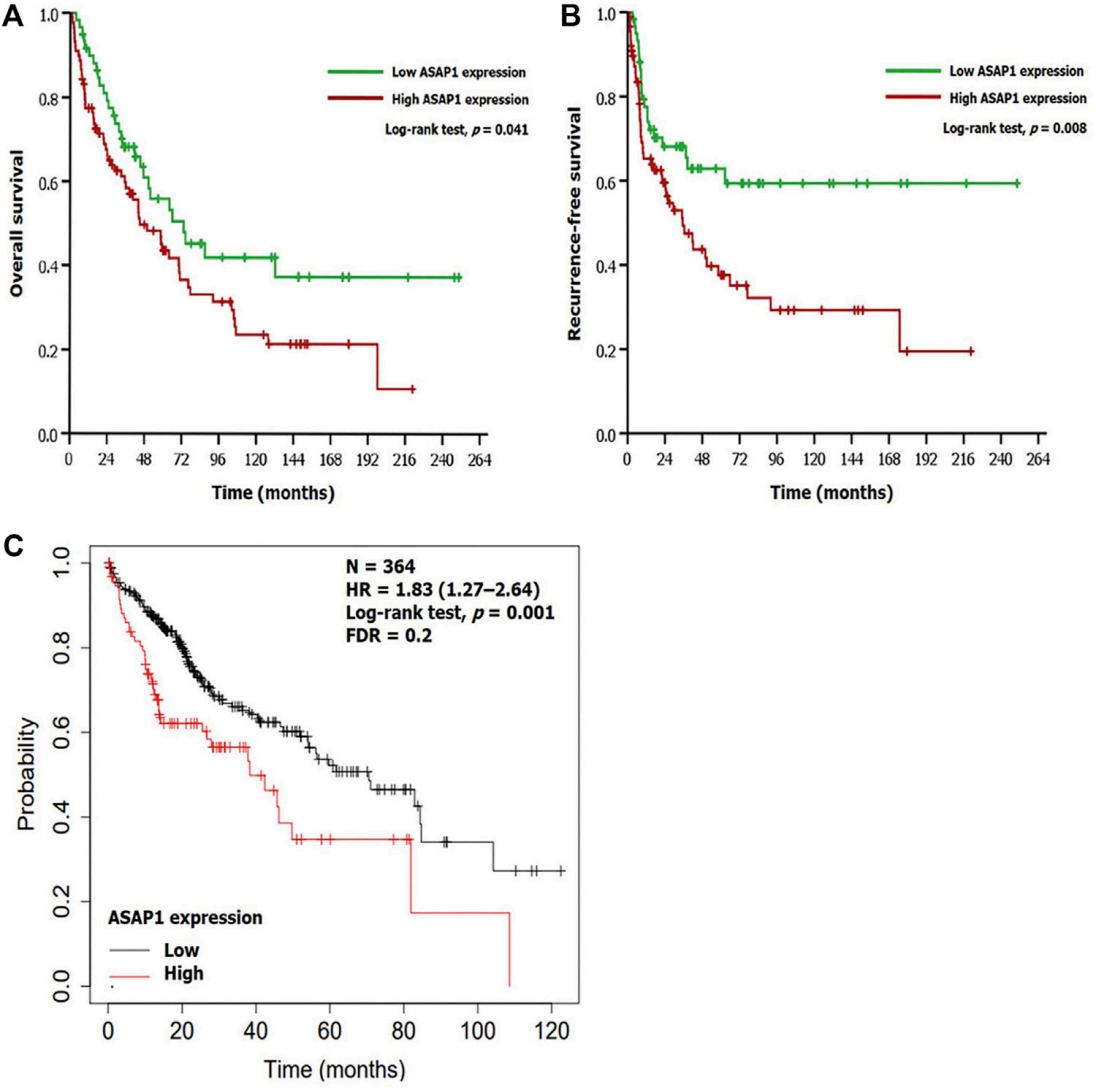
Prognostic implication of ASAP1 expression. **(A,B)** Survival analyses using the Kaplan-Meier method in patients with hepatocellular carcinoma. There was significant difference in overall survival (log-rank test, *p* = 0.041) and recurrence-free survival (log-rank test, *p* = 0.008). **(C)** Web-based analysis using a Kaplan-Meier plotter demonstrated that the group with high ASAP1 mRNA expression showed poor prognosis in patients with hepatocellular carcinoma (*n* = 364; log-rank test, *p* = 0.001).

Based on the Kaplan-Meier method, high ASAP1 expression was significantly associated with shorter OS and RFS (*p* = 0.041 and *p* = 0.008, respectively; Figures 2A,B) in 149 patients with HCC. Univariate analyses for OS and RFS showed ASAP1 expression as a significant prognostic factor (*p* = 0.042 and *p* = 0.009, respectively). Among other clinicopathological factors, higher histological grade (*p* = 0.008), vessel invasion (*p* < 0.001), and higher stage (*p* = 0.001) were associated with shorter OS. Higher histological grade (*p* = 0.003), vessel invasion (*p* < 0.001), perineural invasion (*p* = 0.010), and higher stage (*p* < 0.001) were associated with shorter RFS. In multivariate analyses, short OS and RFS were significantly associated with higher histological grade (*p* = 0.009 and *p* = 0.003, respectively) and higher AJCC stage (*p* = 0.003 and *p* < 0.001, respectively); however, high ASAP1 expression did not remain statistically significant (Table 3). Similar to the results of the present study, the web-based analysis using the KM plotter demonstrated that high mRNA expression of ASAP1 was associated with shorter OS (*p* = 0.001, FDR = 0.2; Figure 2C).

**TABLE 3:** Univariate and multivariate Cox regression analyses for OS and RFS in patients with HCC (*n* = 149).

OS						
Variables	Univariate analysis			Multivariate analysis		
	HR	95% CI	*p-*value	HR	95% CI	*p-*value
ASAP1 expression (low vs. high)	1.587	1.016–2.481	0.042	1.294	0.810–2.067	0.281
Age group (<60 years vs. ≥ 60 years)	1.178	0.744–1.863	0.485			
Tumor size (≤5 cm vs. > 5 cm)	1.351	0.868–2.103	0.183			
Histological grade (grade 1 or 2 vs. grade 3 or 4)	1.796	1.165–2.769	0.008	1.783	1.152–2.759	0.009
Vessel invasion (not identified vs. present)	2.246	1.470–3.432	<0.001			
Perineural invasion (not identified vs. present)	2.958	0.921–9.496	0.068			
Tumor focality (single vs. multiple)	1.121	0.642–1.957	0.687			
Stage^a^ (I or II vs. III)	2.506	1.467–4.282	0.001	2.323	1.324–4.075	0.003
**RFS**						
** Variables**	**Univariate analysis**			**Multivariate analysis**		
	**HR**	**95% CI**	***p-*value**	**HR**	**95% CI**	***p-*value**
ASAP1 expression (low vs. high)	1.975	1.182–3.300	0.009	1.565	0.920–2.662	0.099
Age group (<60 years vs. ≥ 60 years)	0.983	0.580–1.667	0.950			
Tumor size (≤5 cm vs. > 5 cm)	0.966	0.567–1.648	0.900			
Histological grade (grade 1 or 2 vs. grade 3 or 4)	2.108	1.292–3.438	0.003	1.783	1.152–2.759	0.003
Vessel invasion (not identified vs. present)	3.239	2.007–5.228	<0.001			
Perineural invasion (not identified vs. present)	4.819	1.456–15.948	0.010	1.510	0.424–5.375	0.524
Tumor focality (single vs. multiple)	1.626	0.937–2.700	0.097			
Stage* (I or II vs. III)	3.616	2.006–6.518	<0.001	3.263	1.702–6.254	<0.001

*Stage, AJCC 8^th^ edition. Acronyms: OS, overall survival; RFS, recurrence-free survival; HCC, hepatocellular carcinoma; ASAP1, ArfGAP with SH3 domain ankyrin repeat and PH domain 1; HR, hazard ratio; 95% CI, 95% confidence interval.

## Discussion

In the present study, IHC for ASAP1 was performed using human HCC tissues, and each case was classified into a high expression or low expression group. We then assessed the association of ASAP1 expression with clinicopathological features and prognostic significance. High ASAP1 expression was significantly associated with male patients, higher histological grade, vessel invasion, and higher stage. In survival analyses, high ASAP1 expression was associated with a poor prognosis of HCC patients, which was consistent with the result of web-based analysis (KM plotter).

ASAP1 is a downstream molecule of the ARF family and considered an important component of invasion and metastasis as an effector of ARF6 in various tumors (24). Many studies have suggested that ASAP1 expression or co-expression with other signaling molecules has potential as a prognostic biomarker. Müller et al. reported that ASAP1 expression correlates with metastasis and poor OS of patients with colorectal adenocarcinoma (12). Li et al. and Sato et al. showed high ASAP1 expression or co-overexpression of EGFR to be associated with poor prognosis in head and neck squamous cell carcinoma patients (13,14). Hou et al. reported that increased ASAP1 expression was an independent prognostic factor in epithelial ovarian cancer patients (15). Hashimoto et al. revealed that high expression or simultaneous high ASAP1 expression with erythrocyte membrane protein band 4.1 like 5 (EPB41L5) and lysophosphatidic acid receptor 2 (LPAR2) in renal cancer inidicates a poor prognosis (16). Hashimoto et al. reported high ASAP1 expression or co-overexpression of platelet-derived growth factor receptor-beta (PDGFRβ) and EPB41L5 in pancreatic cancer to be associated with poor prognosis (17). Luo et al. showed that high ASAP1 expression and focal adhesion kinase (FAK) was associated with aggressive pathologic features and poor prognosis (18). He et al. and Kinoshita et al. reported that high ASAP1 expression or co-overexpression of guanine nucleotide-exchange protein (GEP100) in breast cancer was associated with tumor recurrence (20, 25).

In several recent studies, the association between the ARF6–ASAP1 pathway and immune evasion in cancer was investigated. Hashimoto et al. showed that programmed death-ligand 1 (PD-L1) expression on the surface of pancreatic cancer cells was significantly reduced after silencing ARF6 and ASAP1 (17). In addition, Tsutaho et al. revealed that high ASAP1 expression in resected tissue of human pancreatic cancer was significantly correlated with high PD-L1 level (26). Horikawa et al. revealed that ASAP1 knockdown significantly reduced carbonic anhydrase IX (CAIX) expression in breast cancer cells and suggested that the ASAP1 pathway is required for the recycling process of CAIX (27). Therefore, ASAP1 has potential as a target or biomarker for predicting responsiveness of immunotherapy in HCC patients considering the associations of ASAP1 with PD-L1 and pH regulatory molecules (28,29).

The exact molecular mechanisms regulating ASAP1 in cancer are unknown. The ASAP1 gene is located at 8q24, and amplification has been reported in several types of tumors. Ehlers et al. reported that mRNA overexpression of ASAP1 in uveal melanoma was significantly correlated with chromosome 8q copy number (9). Similarly, Lin et al. detected additional ASAP1 gene copies and elevated ASAP1 expression at the protein level in prostate cancer (10), and He et al. identified a positive correlation between the copy number gain and ASAP1 gene expression in triple-negative breast cancer (20). Therefore, the gain or amplification of ASAP1 might be partially responsible for the increased ASAP1 expression at the protein level. Further studies are needed in which the molecular mechanism underlying the increased ASAP1 expression in HCC is investigated.

In conclusion, ASAP1 expression was found at the protein level in HCC using IHC. High ASAP1 expression was associated with aggressive clinicopathological characteristics and poor clinical outcomes in patients with HCC. Further studies are needed to confirm the molecular mechanisms regulating ASAP1 expression and its usefulness as a biomarker.

## Data Availability

The original contributions presented in the study are included in the article/Supplementary Material, further inquiries can be directed to the corresponding authors.
